# Alpha-synuclein Toxicity in the Early Secretory Pathway: How It Drives Neurodegeneration in Parkinsons Disease

**DOI:** 10.3389/fnins.2015.00433

**Published:** 2015-11-12

**Authors:** Ting Wang, Jesse C. Hay

**Affiliations:** Division of Biological Sciences, The University of MontanaMissoula, MT, USA

**Keywords:** alpha-synuclein, vesicle trafficking, golgi, ER stress response, neurodegenerative diseases, Parkinson disease, LRRK2, ER to golgi transport

## Abstract

Alpha-synuclein is a predominant player in the pathogenesis of Parkinson's Disease. However, despite extensive study for two decades, its physiological and pathological mechanisms remain poorly understood. Alpha-synuclein forms a perplexing web of interactions with lipids, trafficking machinery, and other regulatory factors. One emerging consensus is that synaptic vesicles are likely the functional site for alpha-synuclein, where it appears to facilitate vesicle docking and fusion. On the other hand, the dysfunctions of alpha-synuclein are more dispersed and numerous; when mutated or over-expressed, alpha-synuclein affects several membrane trafficking and stress pathways, including exocytosis, ER-to-Golgi transport, ER stress, Golgi homeostasis, endocytosis, autophagy, oxidative stress, and others. Here we examine recent developments in alpha-synuclein's toxicity in the early secretory pathway placed in the context of emerging themes from other affected pathways to help illuminate its underlying pathogenic mechanisms in neurodegeneration.

## Introduction

**Figure 1:**
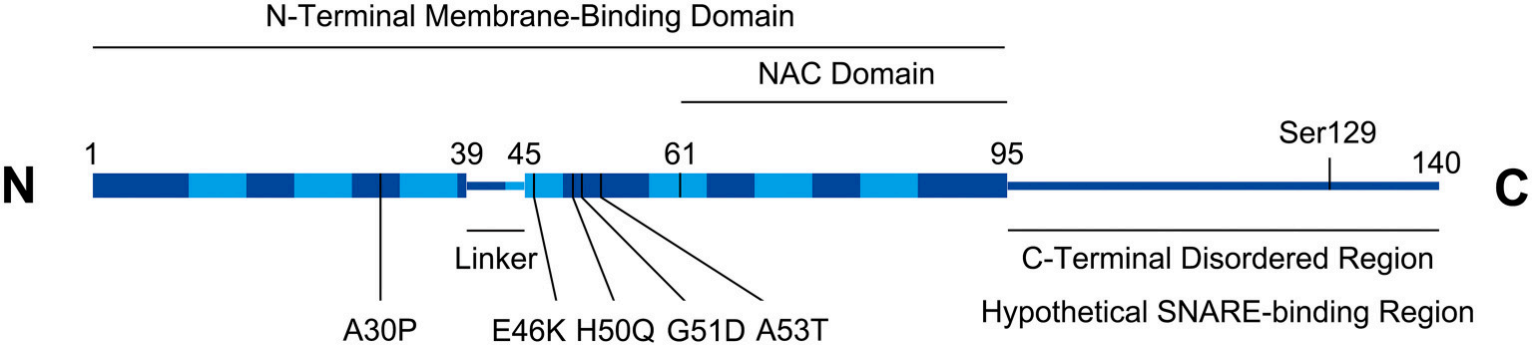
**Alpha-synuclein structural schematic**. Boxed regions represent alpha-helices. Seven repeats (residues 10–15, 21–26, 32–37, 43–48, 58–63, 69–74, and 80–85) in the helical region are depicted with lighter shading. Numbering on figure refers to amino acids. PD-associated missense mutations are indicated. Serine129 is a functionally implicated phosphorylation site. The C-terminal region contains a high density of charged residues (not shown).

Alpha-synuclein (a-syn), a 14 kD cytosolic neuronal protein, has been a major focus of Parkinson's Disease (PD) pathological studies. As shown in Figure 1, a-syn consists of an N-terminal domain that adopts an alpha-helical conformation upon binding to membranes, and a charged C-terminus with multiple phosphorylation sites (Bendor et al., 2013; Snead and Eliezer, 2014). The N-terminal domain of alpha-syn contains seven membrane-interacting repeats, and can either bind the membrane as a long helix, or in a “broken helix” conformation with two helices connected by a linker (Bartels et al., 2010). Alpha-syn monomers can either assemble into tetramers (Bartels et al., 2011) or beta-sheet fibrils that aggregate to form Lewy bodies, a primary indicator of Parkinson's disease (PD) accompanied by neurodegeneration in the Substantia nigra, locus coeruleus, and dorsal vagal nucleus (Kingsbury et al., 2010). Using a prion-like mechanism, normal a-syn is converted to the aggregated form by a-syn molecules that have already been converted (Deleersnijder et al., 2013), a process mediated by the non-amyloid-β component (NAC) region of a-syn, comprising residues 61–95. Alpha-syn gene duplication, triplication, and several dominant mutations, including A30P, E46K, H50Q, and A53T cause familial PD (Polymeropoulos et al., 1997; Zarranz et al., 2004; Khalaf et al., 2014).

The alpha-, beta-, and gamma-synuclein genes are somewhat functionally redundant (Greten-Harrison et al., 2010; Ninkina et al., 2012; Vargas et al., 2014), which probably accounts for the lack of a significant phenotype from a-syn single knockout (Abeliovich et al., 2000; Cabin et al., 2002). Synuclein triple knockout, however, alters synapse structure and leads to age-dependent neuronal dysfunction in mice (Greten-Harrison et al., 2010). Alpha-syn participates in the maintenance of the presynaptic vesicle pool as well as the release of neurotransmitter (Murphy et al., 2000; Cabin et al., 2002; Ben Gedalya et al., 2009; Nemani et al., 2010; Cheng et al., 2011).

The neuronal toxicity of alpha-synuclein over-expression or mutation appears to involve a wide range of pathways and cellular functions including ER-to-Golgi transport, Golgi homeostasis, presynaptic trafficking, endocytosis, autophagy, the ubiquitin-proteasome system, ER and oxidative stress (Snead and Eliezer, 2014). This review focuses primarily on the dysfunction of alpha-synuclein and pathogenically related proteins in the early secretory pathway.

## Physiological functions

Although present in various compartments of the cell, alpha-synuclein accumulates at the synapse (Maroteaux et al., 1988; Kahle et al., 2000), and is recruited to synaptic vesicles through interactions with specific lipids and trafficking machinery (Fortin et al., 2004; Burré et al., 2010; Diao et al., 2013). In addition, a-syn is capable of sensing membrane curvature and lipid composition, with a preference for synaptic vesicle's small shape, high curvature, and charged lipid head groups (Pranke et al., 2011; Bendor et al., 2013). The Rab family of small GTPases appear to facilitate the recruitment process (Lee et al., 2011; Chen et al., 2013) as does C-terminal phosphorylation (Oueslati et al., 2010; Paleologou et al., 2010).

One emerging consensus is that a-syn, perhaps in cooperation with cysteine string protein (CSP) (Chandra et al., 2005), acts as a SNARE chaperone to potentiate SNARE assembly for fusion (Burré et al., 2010). Interactions between a-syn and synaptic SNAREs are established, and a-syn can promote SNARE-mediated vesicle fusion in a manner that requires both lipid and SNARE interactions. The precise step modulated by a-syn seems to lie slightly upstream of final SNARE assembly during clustering, docking, and/or SNARE priming for assembly (Diao et al., 2013; Burré et al., 2014).

## Dysfunctions I: the synapse

Alpha-syn's potential dysfunctions at its native site, the synapse, have been intensely researched but will be briefly treated here. Confusingly and despite the positive functions in docking and fusion described above, a-syn has been shown to inhibit these steps as well. Large alpha-syn oligomers potently inhibit vesicle fusion (Choi et al., 2013, 2015) and soluble, non-aggregated a-syn also inhibits at higher concentrations (Lai et al., 2014). In some conditions, a-syn's interactions with lipids alone may inhibit SNARE-mediated fusion (DeWitt and Rhoades, 2013; Lai et al., 2014). Alpha-syn synaptic function vs. dysfunction is likely very sensitive to concentration and the aggregation state of a-syn species, keeping in mind that conversion to pathogenic forms will also deplete cells of the functional form. However, it remains to be established whether a-syn synaptic dysfunction is a major driver of neurodegeneration in PD.

## Dysfunctions II: the ER-Golgi axis

The mechanism of a-syn toxicity leading to neurodegeneration is very complex, multi-pronged, and highly integrated with cellular stress pathways. Most likely, a-syn oligomers directly intoxicate several cellular membrane-dependent pathways, each of which causes damage and activation of unsuccessful stress responses leading to apoptosis.

Evidence has mounted that one of the initiating insults results from a-syn's disruption of ER-to-Golgi transport, the first and rate-limiting step in the biosynthetic secretory pathway. This effect was first identified in a yeast screen for genetic suppressors of a-syn toxicity (Cooper et al., 2006). Surprisingly, the most efficient group of suppressors were membrane trafficking proteins, including Ypt1p/rab1, which coordinates ER/Golgi vesicle tethering; and Ykt6p, a SNARE that mediates ER/Golgi-related fusions. That the most successful suppressors were ER/Golgi trafficking machinery suggested that this pathway is deeply involved in a-syn toxicity. Follow-up studies by multiple groups have demonstrated potent inhibition of ER-to-Golgi transport by physiologically relevant over-expression levels of a-syn in normal rat kidney cells, HeLa cells, neuroendocrine PC12 cells, and dopaminergic SH-SY5Y cells (Thayanidhi et al., 2010; Winslow et al., 2010; Oaks et al., 2013). The evidence supports a potent inhibition of the secretory pathway by mild over-expression of wildtype a-syn and at even lower concentrations by PD-associated mutants. The locus of a-syn inhibition appears to be post- ER budding, perhaps through interference with rab- and SNARE-dependent COPII vesicle tethering and/or fusion (Gitler et al., 2008; Thayanidhi et al., 2010). Interestingly, superoxide dismutase-1 (SOD1) mutants that cause neurodegeneration in amyotrophic lateral sclerosis (ALS) have also been shown to inhibit ER-to-Golgi transport in neurons resulting in ER stress that can be ameliorated by over-expression of ER/Golgi vesicle machinery (Atkin et al., 2014). Is there a common feature of aggregated cytosolic proteins that is fundamentally linked to ER export?

Normal neurons may thus be treading a delicate balance in which they must express a-syn at a sufficient level to promote synaptic trafficking yet below a threshold where it inhibits biosynthetic trafficking and causes stress in the cell body. How can a-syn be on the one hand a chaperone for facilitating SNARE-dependent synaptic vesicle docking/fusion, and on the other hand a disruptor of mechanistically similar membrane docking/fusion events in the early secretory pathway? One key could be the membrane binding properties of a-syn, which cause it to preferentially interact with post-Golgi, highly curved, charged membranes (Pranke et al., 2011) and lead the N-terminal region to adopt helical structure and self-aggregate (Burré et al., 2014). If these membrane interactions affect a-syn interactions with SNAREs and rabs, then perhaps only post-Golgi lipid compositions support positive effects. Conversely, a-syn molecules that are not properly primed by lipid interactions would fail to exert positive effects, or even negatively affect SNARE assembly on pre-Golgi membranes. Both SNAREs and a-syn undergo binding-induced helix formation, which could make their coordinated assembly extremely context dependent. It would be very useful to know whether a-syn can have different effects at distinct membrane trafficking steps under the same expression conditions in the same cell.

Recently, the link between ER/Golgi trafficking and PD became even stronger. Mutations in Leucine-rich repeat kinase 2 (LRRK2) are the most frequent genetic lesions associated with PD (Kumari and Tan, 2009). Strong genetic and possible physical interactions between a-syn and LRRK2 suggest that they may intoxicate a common pathway (Lin et al., 2009; Qing et al., 2009; Guerreiro et al., 2013). While the precise functions of LRRK2 are still emerging, they appear to be converging on ER export and secretory trafficking. Interactions between LRRK2 and the ER exit site (ERES) scaffold sec16 led to the discovery that LRRK2 is required for ERES assembly and efficient ER cargo exit (Cho et al., 2014). Sec16 is believed to work in concert with Sar1 GTPase to facilitate the assembly of COPII coat components (Watson et al., 2006). One of the common PD-associated LRRK2 mutants, R1441C, disrupts targeting of sec16 to ERES. In parallel, another mechanism by which LRRK2 may impact secretion has emerged. Screens for LRRK2 interactions identified BCL2-associated athanogene 5 (BAG5), Rab7L1, and cyclin-G–associated kinase (GAK) as binding partners. These proteins appeared to form a single complex targeted to the TGN that positively regulates TGN turnover (Beilina et al., 2014). Over-expression of any of these proteins alone, or expression of PD-associated mutations of LRRK2, led to ablation of TGN markers, and presumably the organelle itself, via an autophagy mechanism (Beilina et al., 2014). Severe down-regulation of the TGN would disrupt secretory trafficking and ultimately lead to intracellular accumulation of secretory cargo at multiple steps. Elucidating the multiple cellular functions of LRRK2 is essential for understanding the secretory pathology of PD. Another development was that rab39b lesions are associated with early-onset Parkinsonism (Wilson et al., 2014). Although still very early, it appears that rab39b and protein interacting with C-kinase 1 (PICK1) regulate ER-to-Golgi transport of synaptic machinery (Mignogna et al., 2015). To summarize, genetic lesions in a-syn, LRRK2 and other genes–including the most potent and frequent lesions causing PD–may initiate neuronal stress by disrupting early steps in secretion. It is urgent to elucidate the complex relationship between ER/Golgi trafficking, neuronal stress, and PD.

Alpha-syn probably disrupts intra-Golgi and post-Golgi secretory trafficking in addition to ER-to-Golgi trafficking. Alpha-syn interacts with Rab8 (Golgi), Rab3a (post-Golgi), and Rab5 (early endosomal) in a-syn A30P transgenic mice (Dalfó et al., 2004); in addition, Rab8 (Golgi), Rab3a (post-Golgi), and rab11 (recycling endosomal) overexpression rescue alpha-syn's toxicity (Cooper et al., 2006; Gitler et al., 2008; Yin et al., 2014; Breda et al., 2015). That a-syn toxicity involves simultaneous block of multiple steps in the biosynthetic secretory pathway is also supported by the potent suppression of a-syn transport blocks by ykt6 (Cooper et al., 2006; Thayanidhi et al., 2010), an R-SNARE that can participate in multiple fusogenic SNARE complexes. One secretory cargo of particular importance to dopaminergic neurons is the dopamine transporter (DAT); indeed DAT accumulates in microsomal fractions upon a-syn over-expression and dopamine uptake is consequently inhibited (Oaks et al., 2013). However, as outlined below, ER accumulation, as opposed to lack of proper delivery of secretory and membrane proteins may drive neurodegeneration even more substantially.

## Dysfunctions III: ER stress

Alpha-syn over-expression causes ER stress (ERS) and activation of the unfolded protein response (UPR) in cellular models (Smith et al., 2005), which causes apoptosis if unresolved. Accumulating evidence from many systems and approaches indicates that UPR plays a rate-limiting role in the progression of neurodegeneration and a-syn pathology (Mercado et al., 2013). UPR is activated in PD neurons and UPR modulation protects or potentiates PD progression. Low levels of UPR protects neurons from a-syn toxicity, presumably by inducing chaperones and secretory machinery to counteract ERS (Fouillet et al., 2012; Valdés et al., 2014). Similarly, prevention of the adaptive response or inducing acute ERS accelerates a-syn-associated neuronal death (Colla et al., 2012a; Salganik et al., 2015). Phosphorylation of Ser129 on alpha-syn appears to be important for triggering UPR (Colla et al., 2012a). Interestingly, a-syn intoxication does not activate all three classical branches of the UPR equally, which could help explain why this particular ERS cannot be adapted to. Work in PD models found that induction of chaperones and CHOP occurs without phosphorylation of eIF2α by PERK (Colla et al., 2012a), perhaps indicating that adaptive and pro-apoptotic responses are out of balance. Other studies indicate that activation of IRE1/XBP1 is key to neuronal survival in PD, and that this particular UPR branch may be selectively important in neurons of the substantia nigra (Valdés et al., 2014). Still other work finds that a-syn toxicity involves the imbalanced outcomes of increased chaperones accompanied by decreased activation of the ATF6 pathway (Credle et al., 2015). A compelling correlate of this hypothesis is that activation of the ATF6 pathway requires ER/Golgi transport, and ATF6 export from the ER is potently inhibited by a-syn (Credle et al., 2015).

Precisely how does a-syn cause ERS in the first place? The most obvious mechanism would be inhibition of ER/Golgi transport and/or later steps in the biosynthetic secretory pathway, leading to ER overload. The finding that over-expression of secretory rabs and other machinery alleviates a-syn toxicity is consistent with ERS originating from a trafficking defect (Gitler et al., 2008; Yin et al., 2014). However, ER/Golgi transport could also be viewed as a relief valve for ERS caused by perturbation of other ER functions. In addition, some of the secretory machinery whose expression rescues stress, rab1a for example, may also more directly regulate UPR events or autophagy induction (Winslow et al., 2010; Chua and Tang, 2013).

Evidence exists for several modes by which a-syn could initiate ERS through mechanisms distinct from ER/Golgi trafficking. For example, it has been suggested that a-syn may find its way inside the lumen of the ER, where it could aggregate with chaperones to cause ERS (Colla et al., 2012b). A mechanism for a-syn to cross the ER membrane has not been demonstrated. One speculation could be uptake of extracellular a-syn aggregates by endocytosis followed by retrograde vesicular transport to the ER lumen, as in the case of certain bacterial toxins (Spooner and Lord, 2012). A completely distinct mechanism by which a-syn may trigger ERS is by inhibition of ER associated degradation (ERAD), which would cause buildup of excess unfolded proteins. Homocysteine-induced ER protein (Herp) is overexpressed in PD and is a component of Lewy bodies (Slodzinski et al., 2009) whose knockdown sensitizes PC12 cells to ERS-induced apoptosis and a-syn toxicity (Belal et al., 2012). Herp plays an essential role in ERAD (Schulze et al., 2005), particularly ERAD-dependent turnover of ER calcium channels (Belal et al., 2012). Herp inhibition or aggregation in PD could lead to general ERAD inhibition or ER calcium depletion, either of which could cause ERS. ERAD relies upon the ubiquitin-proteasome system (UPS) for the disposal of ER-dislocated substrates, and there are many links between the UPS and PD, only discussed here as it relates to ERS. Several E3 ubiquitin ligases, for example parkin, HRD1 and Rsp5/Nedd4, can protect cells from a-syn intoxication and ERS (Tsai et al., 2003; Omura et al., 2013; Tardiff et al., 2013). The mechanism by which ubiquitin ligases protect neurons from ERS is unclear. One possibility is that the UPS contributes to the removal of specific stress-causing membrane proteins such as parkin-associated endothelin receptor-like receptor (Pael-R) that have been implicated in PD pathology (Kitao et al., 2007; Omura et al., 2013). Parkin also triggers mitophagy when recruited to defective mitochondria by the voltage sensor PINK (Kroemer et al., 2010). But there could also be more fundamental link(s) whereby the UPS promotes ER homeostasis, for example through a direct regulatory role in the initiation of UPR, ERAD, or ER-phagy. This could help explain why multiple neurodegenerative diseases involving aggregated cytosolic proteins result in UPR–a process conventionally viewed as initiated in the ER lumen.

## Dysfunctions IV: Golgi homeostasis

Golgi fragmentation is commonly observed in PD as well as other neurodegenerative diseases (Gonatas et al., 2006; Fan et al., 2008; Bexiga and Simpson, 2013). Overexpression of a-syn in cultured mammalian cell lines and neurons can lead to Golgi fragmentation; formation of a-syn prefibrillar aggregates correlates tightly with Golgi and microtubule disruption (Gosavi et al., 2002; Lee et al., 2006). Alpha-syn accumulation in yeast results in mislocalization of Golgi markers and aggregation of secretory vesicles (Soper et al., 2008, 2011). However, the functional and pathological significance of Golgi disruption remains unclear. In some cases, Golgi disruption seems to precede microtubule disruption and inhibition of ER/Golgi transport (Rendón et al., 2013), implying that Golgi disruption is not a result of the trafficking block, and could represent an independent branch of the pathology. In other cases the Golgi disruption seems to accompany and be due to the ER/Golgi transport inhibition (Coune et al., 2011) or be absent during transport inhibition (Thayanidhi et al., 2010).

The lack of a consistent correlation between ER/Golgi transport inhibition and Golgi disruption makes the role of Golgi homeostasis in PD hard to evaluate. However, an intriguing idea is that Golgi dispersal may be a means to propagate an a-syn-caused Golgi-based stress signal. Increased neuronal firing activity causes reversible Golgi fragmentation, suggesting it may be part of a homeostatic stress signaling mechanism (Thayer et al., 2013). Furthermore, inhibition of Golgi dispersal through over-expression of Golgi tethering proteins can reduce the toxicity of chemically-induced ER or oxidative stress (Nakagomi et al., 2008). How Golgi fragmentation is initiated in response to a-syn remains unclear, but signaling mechanisms mediating other stress-related Golgi fragmentation have been outlined. For example, the small GTPase ADP-ribosylation factor 4 (ARF4) initiates Golgi dispersal in response to pathogens and chemical insults (Reiling et al., 2013), and Cdk5 kinase phosphorylation of Golgi matrix protein 130 (GM130) may mediate Golgi dispersal in neurons challenged with beta-amyloid (Sun et al., 2008). It would be exciting to test whether blockade of Golgi-dispersing signals can relieve a-syn toxicity.

## The endolysosomal system and alpha-synuclein clearance

The importance of the endolysosomal system in a-syn toxicity cannot be overstated but will only be briefly introduced. The clearance of a-syn largely depends upon the endosome-lysosome pathway (Flower et al., 2007; Lee et al., 2008; Liu et al., 2009; Alvarez-Erviti et al., 2011). This process appears to be Rab11a-dependent (Liu et al., 2009), which likely explains Rab11a's involvement in synucleinopathy (Breda et al., 2015). Alpha-syn over-expression appears to interfere with endosomal trafficking (Soper et al., 2008, 2011; Volpicelli-Daley et al., 2014), and this likely exacerbates a-syn toxicity by potentiating a-syn accumulation. In addition, impaired lysosomal activity could contribute to PD (van Dijk et al., 2013).

Alpha-syn accumulation triggers macroautophagy, the process by which cytosolic proteins and organelles are imported into lysosomes. ERS triggered by a-syn accumulation probably contributes to autophagy activation since the PERK and ATF6 UPR signaling arms induce autophagy genes (He and Klionsky, 2009; Kroemer et al., 2010). However, aggregated or over-expressed alpha-syn inhibits autophagy and results in less efficient clearance, more accumulation and greater cell-to-cell spread of a-syn pathology (Winslow et al., 2010; Lee et al., 2013; Tanik et al., 2013).

## Oxidative stress

Oxidative stress is another outcome of alpha-syn accumulation, and is closely associated with disruption of ER proteostasis (Exner et al., 2012). Similar to ERS, mitochondrial dysfunction is present in many neurodegenerative diseases (Lionaki et al., 2015), and will be important in disease intervention. Still, a key question remaining is whether the pathological oxidative stress is a downstream manifestation of other stresses, for example perturbed ER calcium or reduced autophagic clearance of defective mitochondria (Chigurupati et al., 2009; Su et al., 2010), or does it belie a more fundamental relationship with alpha-synuclein?

## Conclusion

**Figure 2:**
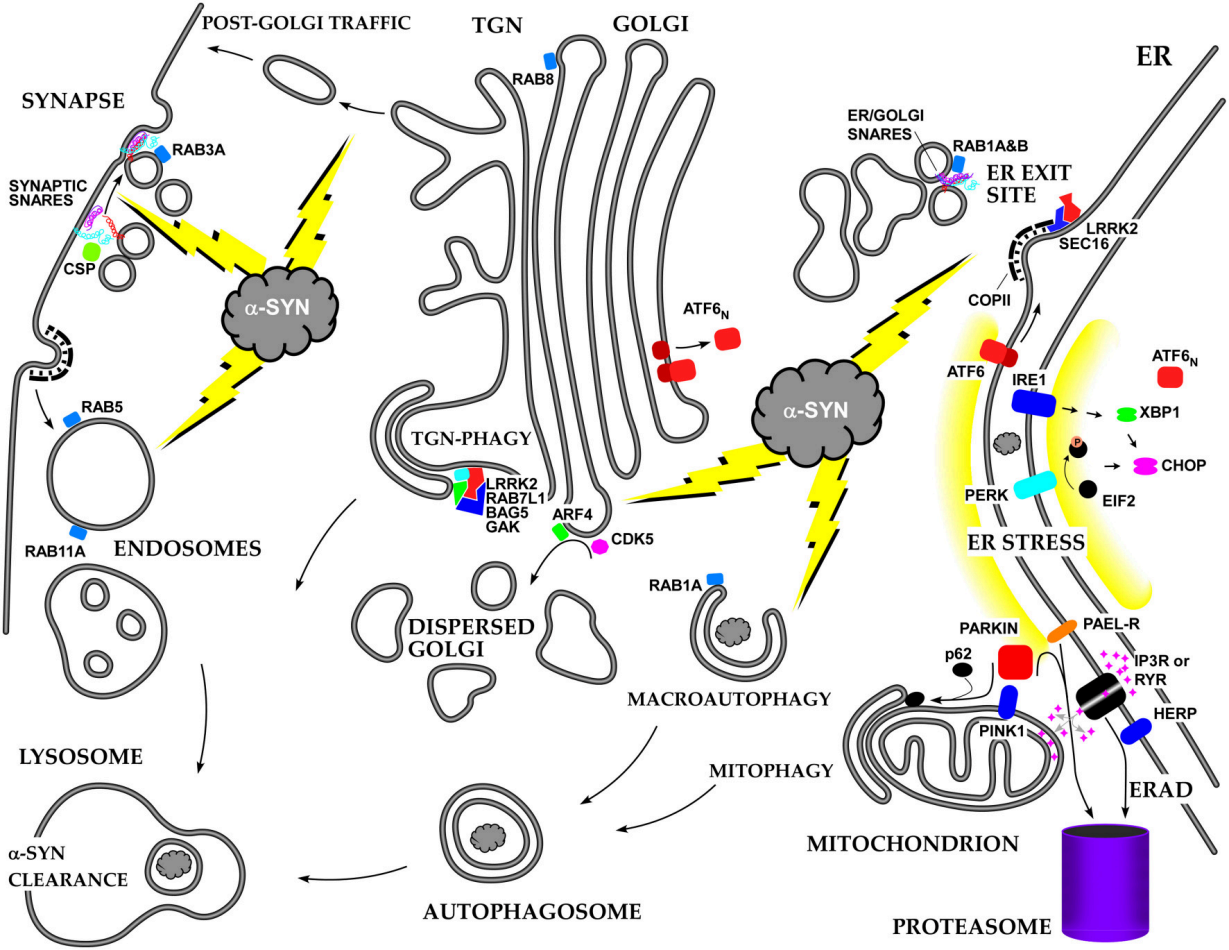
**Summary of the roles of alpha-synuclein and other PD-associated proteins in the secretory pathway and the resulting stress responses**.

We have briefly summarized the multifaceted effects of alpha-syn on cellular membrane trafficking, with special emphasis on the ER-Golgi axis (see Figure 2). Many more studies will be required to unravel all of the mechanisms of alpha-syn toxicity, and the early secretory pathway will likely remain a major focus. One of the major issues to resolve is how a-syn manages to play positive as well negative roles in membrane trafficking due to its concentration, aggregation state, and SNARE- and lipid-binding properties. Another major issue is how a-syn triggers its unique kind of ERS and how this stress is modulated by ER/Golgi transport, ERAD, and the ubiquitin-proteasome system. In addition, it should be resolved whether there is a common pattern of dysfunction in the early secretory pathway lying at the heart of multiple neurodegenerative diseases.

### Conflict of interest statement

The authors declare that the research was conducted in the absence of any commercial or financial relationships that could be construed as a potential conflict of interest.
